# Limited heat tolerance in a cold-adapted seabird: implications of a warming Arctic

**DOI:** 10.1242/jeb.242168

**Published:** 2021-07-07

**Authors:** Emily S. Choy, Ryan S. O'Connor, H. Grant Gilchrist, Anna L. Hargreaves, Oliver P. Love, François Vézina, Kyle H. Elliott

**Affiliations:** 1 Department of Natural Resource Sciences, McGill University, Ste Anne de Bellevue, QC, Canada H9X 3V9; 2 Département de Biologie, Chimie et Géographie, Université du Québec à Rimouski, Rimouski, QC, Canada 95L 3A1; 3 Groupe de recherche sur les environnements nordiques BORÉAS, Institut nordique du Québec, Université du Québec à Rimouski, Rimouski, QC, Canada 95L 3A1; 4 Centre d’études Nordiques, Université du Québec à Rimouski, Rimouski, QC, Canada 95L 3A1; 5 Centre de la Science de la Biodiversité du Québec, Université du Québec à Rimouski, Rimouski, QC, Canada 95L 3A1; 6 National Wildlife Research Centre, Environment and Climate Change Canada, 1125 Colonel By Dr, Ottawa, ON, Canada K1S 5B6; 7 Department of Biology, McGill University, Montreal, QC, Canada H3G 0B1; 8 Department of Integrative Biology, University of Windsor, Windsor, ON, Canada N9B 3P4

**Keywords:** Arctic climate change, Evaporative cooling efficiency, Evaporative water loss, Thick-billed murres, Heat stress, Seabirds

## Abstract

The Arctic is warming at approximately twice the global rate, with well-documented indirect effects on wildlife. However, few studies have examined the direct effects of warming temperatures on Arctic wildlife, leaving the importance of heat stress unclear. Here, we assessed the direct effects of increasing air temperatures on the physiology of thick-billed murres (*Uria lomvia*), an Arctic seabird with reported mortalities due to heat stress while nesting on sun-exposed cliffs. We used flow-through respirometry to measure the response of body temperature, resting metabolic rate, evaporative water loss and evaporative cooling efficiency (the ratio of evaporative heat loss to metabolic heat production) in murres while experimentally increasing air temperature. Murres had limited heat tolerance, exhibiting: (1) a low maximum body temperature (43.3°C); (2) a moderate increase in resting metabolic rate relative that within their thermoneutral zone (1.57 times); (3) a small increase in evaporative water loss rate relative that within their thermoneutral zone (1.26 times); and (4) a low maximum evaporative cooling efficiency (0.33). Moreover, evaporative cooling efficiency decreased with increasing air temperature, suggesting murres were producing heat at a faster rate than they were dissipating it. Larger murres also had a higher rate of increase in resting metabolic rate and a lower rate of increase in evaporative water loss than smaller murres; therefore, evaporative cooling efficiency declined with increasing body mass. As a cold-adapted bird, murres' limited heat tolerance likely explains their mortality on warm days. Direct effects of overheating on Arctic wildlife may be an important but under-reported impact of climate change.

## Abstract (precis)

**Summary:** Thick-billed murres have limited heat tolerance, displaying low evaporative cooling efficiencies and increases in physiological traits associated with heat stress at low air temperatures relative to desert birds.

## INTRODUCTION

Climate change is warming the Arctic at approximately twice the global rate (Anisimov et al., 2007; McBean et al., 2005). In the Canadian Arctic, mean annual air temperature has increased by ∼2.3°C from 1948 to 2016, and could increase by an additional 7.8°C by 2100 under high-emission scenarios (Zhang et al., 2019). This warming may have severe effects on cold-adapted homeothermic endotherms (i.e. organisms that actively maintain relatively constant body temperatures through metabolic heat production). Several studies have highlighted the indirect effects of warming on Arctic wildlife, such as compositional shifts in the prey base (Gaston and Hipfner, 1998; Gaston et al., 2005; Yurkowski et al., 2018), earlier breeding phenology and shifts in the timing of migration (Chmura et al., 2020; Clairbaux et al., 2019; Le Corre et al., 2017). In contrast, the direct effects of warming on the physiology and behaviour of Arctic endotherms has been observed but less studied (Gaston et al., 2002).

There is growing evidence that the heat tolerance limits of endotherms have ecological consequences (Rezende and Bacigalupe, 2015). In birds, which maintain their core body temperature at levels higher than in mammals (41°C versus 37°C), heat waves have caused mass mortality events (McKechnie et al., 2012) and reproductive failures (Bolger et al., 2005; Boersma and Rebstock, 2014). The forecasted increase in heat wave frequency is predicted to cause declines in select avian populations (Conradie et al., 2019; McKechnie and Wolf, 2010). However, as most avian heat tolerance studies have focused on desert birds (Gerson et al., 2014; Smit and Mckechnie, 2015; Whitfield et al., 2015), less is known about heat tolerance in Arctic birds. Recent evidence suggests that an Arctic passerine may be limited in its capacity to withstand even moderately high air temperatures (O'Connor et al., 2021). As larger birds have proportionally less surface area to volume ratios, and therefore less surface to dissipate heat, they may be even more sensitive to heat stress.

Diving Arctic seabirds are exposed to a range of environmental temperatures, resulting in an additional thermoregulatory challenge; they must avoid heat stress during nesting, yet must minimize heat loss while diving under icy waters for their prey. One such species is the thick-billed murre (*Uria lomvia*; hereafter ‘murres’). Murres have a circumpolar distribution (Frederiksen et al., 2016) and forage in waters typically colder than 8.0°C (Gaston et al., 2005).

Murres face several indirect impacts of climate change (Gaston et al., 2003, 2005, 2009), but mounting evidence suggests they will also be directly affected by warming during the breeding season (Gaston and Elliott, 2013; Gaston et al., 2002). Murres have been observed displaying heat dissipation behaviours (heavy panting and wing spreading), and have died on their nests as a result of heat stress and mosquito parasitism during calm days with maximum air temperatures as low as 22°C (Gaston and Elliott, 2013; Gaston et al., 2002). Murres' black back feathers have also been reported to reach temperatures as high as 46°C in full sun at ambient temperatures no greater than 23°C (Gaston et al., 2002). Despite evidence that murres are sensitive to heat, there has been no research that has linked increasing air temperatures to heat stress, which is important for predicting the effects of forecasted Arctic warming.

To examine physiological responses to heat stress in murres, we exposed birds to increasing air temperatures (*T*_a_) and measured responses in: (1) body temperature (*T*_b_); (2) resting metabolic rate (RMR); (3) evaporative water loss (EWL); and (4) evaporative cooling efficiency (the ratio of evaporative heat loss to metabolic heat production: EHL/MHP; Lasiewski et al., 1966). To ascertain the onset of heat stress, for each measurement we identified inflection points, which represent a sudden change in the trait due to increasing *T*_a_. We predicted that as a large (∼1 kg) seabird with high metabolic rates relative to its size (Gabrielsen et al., 1988) that is adapted for foraging in cold waters, murres would exhibit inflection points at low *T*_a_ and tolerate low maximum *T*_a_ relative to smaller and more heat tolerant species. Therefore, we predicted murres would also have higher costs of thermoregulation and display large increases in RMR relative to birds of the same body size from lower latitudes.

## MATERIALS AND METHODS

### Study site, temperature and gas exchange measurements

All bird handling was approved by the animal care committee at McGill University and conducted under scientific permits from Environment and Climate Change Canada (Banding Permit 10892, Scientific Permit NUN-SCI-16-03) and the Government of Nunavut (2019-021). We studied thick-billed murres, *Uria lomvia* (Linnaeus 1758), between 26 June and 7 August 2019, from the ‘West colony’ on Coats Island in northern Hudson Bay, NU, Canada (62°57'N, 82°00'W). The West colony has approximately 30,000 breeding pairs of murres nesting on cliffs. Birds lay eggs around mid-June, and chicks fledge by early to mid-August (Gaston et al., 1994, 2012). We captured incubating adult murres (*n=*10) using noose polls. We were restricted in the number of murres we could capture by logistical constraints associated with the challenges of working in a remote Arctic location (no electricity, limited supplies, poor weather, etc.) and were unable to return in 2020 because of travel restrictions into Nunavut in response to COVID-19. We measured body mass (*M*_b_) using a CS Series Ohaus portable scale (2000±1 g), by placing individual birds head-first into a tared cylinder (which secured their wings and held them still) on the scale and waited until the mass stabilized.

To determine RMR and rates of EWL of individual murres, we measured oxygen consumption rate (*V̇*_O_2__; ml min^−1^) and water vapour pressure (WVP; kPa), using flow-throw respirometry. Immediately following capture, individual murres were placed in a sealed Plexiglas metabolic chamber (42×42×41 cm) at the Coats Island field station. The chamber was fitted with a mesh base; guano fell through and into approximately 1 cm of mineral oil covering the base of the chamber, which prevented evaporation from guano affecting WVP measurements. We placed the chamber inside a temperature-controlled and insulated box fitted with a Peltier heating unit (model T35 DC-S, Mobicool International, Zhuhai, China) and a Watercarbon Tech Era carbon fibre seat heater (Henan, China). We monitored and regulated *T*_a_ inside the box using a digital thermostat (ITC-1000F, Shenzhen Inkbird Technology Co., Shenzhen, China). We measured *T*_a_ inside the metabolic chamber using a thermistor probe (Sable Systems, Las Vegas, NV, USA) inserted through a small hole in the chamber and sealed with putty (Gorilla all-purpose epoxy) and connected to a Field Metabolic System (Sable Systems). *T*_b_ was measured using a thermocouple probe (TC-1000 Type-T Thermometer) inserted approximately 3 cm in the cloaca and secured with electrical tape. The thermocouple was connected to a Sable Systems thermocouple meter (model TC-1000) that measured *T*_b_ every second. Although the thermocouple probe fell out prematurely at *T*_a_=22.3°C in one murre, we were able to obtain complete *T*_b_ measurements on 9 birds.

We pushed atmospheric air into the metabolic chamber using an air pump (model ECOair 7, EcoPlus Commercial Air Pump). Air was scrubbed of water vapour and CO_2_ by passing the air stream through columns of Drierite (W.A. Hammond, Drierite Co. Ltd, Xenia, OH, USA) and soda lime connected in series. Once scrubbed, the airstream was split into a baseline channel that went directly to the analysers and a second channel that went to the chamber. Baseline flow rates were controlled using a needle valve (AS4200F, SMC, Tokyo, Japan), whereas chamber flow rates were controlled with an OMEGA mass flow controller (calibrated 18 January 2019; model FMI-100-MKC-2C, Norwalk, CT, USA). We maintained flow rates at 2630 ml min^−1^. These flow rates produced a mean maximum chamber dew point of 14.3°C (range 3.6–18.0°C). The maximum absolute humidity was 14.36 g m^−3^ at *T*_a_=38.4°C.

We subsampled incurrent air from the baseline channel and excurrent air from the metabolic chamber by manually switching between them using the Field Metabolic System, which pulled air and first measured WVP. Within the same system, the airstream was then scrubbed of water vapor and CO_2_ for the measurement of O_2_ consumption. All tubing connecting the system was Bev-A-Line (Thermoplastic Processes Inc., Warren, NJ, USA). We digitized voltage outputs from all the analysers using a Sable Systems Universal Interface (model UI-2) and logged analyser outputs at a sampling rate of 5 s (0.2 Hz) with Expedata software (v.1.9.14, Sable Systems).

### Experimental protocol

We placed murres in the chamber at approximately 21:00 h, within 5 min of capture, at a temperature within their thermoneutral zone (mean±s.e.m.: 18.6±0.1°C; range: 15.7 to 20.4°C; Gabrielsen et al., 1988; Elliott et al., 2013a) and held overnight for 14.65±1.4 h. All heat tolerance measurements took place immediately the following day between 06:00 h and 18:00 h (note however that previous studies have found no diurnal rhythm in the RMR of murres; Elliott et al., 2013a: from 04:30 h to 20:30 h on Coats Island; Gabrielsen et al., 1988: from 09:00 h to 03:00 h at NyÅlesund, Svalbard). Starting in the thermoneutral zone at *T*_a_≈18.8°C, we exposed individual murres to a ramped profile of increasing *T*_a_ by approximately 2°C increments. Once the chamber *T*_a_ stabilized at ±1°C for approximately 2 min, we recorded data (*V̇*_O_2__, WVP, *T*_b_) for approximately 30 min before increasing *T*_a_. A 10–25 min baseline was recorded at the beginning and end of each run, and in between measurements while adjusting the chamber temperature. To observe whether birds remained calm while in the chamber, we monitored their behaviour using a SmotecQ dome infrared camera (model DF-3500-AHD 1080P) and video capture software (ArcSoft ShowBiz, v.3.5.15.68). Following the protocol of Whitfield et al. (2015), we ceased experiments if birds exhibited continuous erratic behaviour such as pacing, flapping or escape behaviour, or an uncontrolled increase in *T*_b_ to 45°C. As the maximum *T*_b_ of murres was 43.3°C, we monitored continuous escape behaviour, which typically lasted for more than 2 min at the maximum *T*_a_ measured. After the run was terminated, birds were re-weighed and brought immediately outside to cool down. Once their behaviour and breathing appeared normal, murres were brought back to their nesting site and released into the wild. No adverse effects were observed and all birds were witnessed to have returned to their nesting site. 

### Data analysis

To examine the physiological responses to increasing *T*_a_, we analysed *V̇*_O_2__ and WVP data with Expedata 1.9.14. We first corrected for the time lag in the O_2_ and water vapour traces by using the lag correction function in Expedata. Next, we used a *z*-transformation (Bartholomew et al., 1981) to correct for chamber volume relative to the flow rate. We used a Catmull–Rom spline correction applied to baselines to correct for drift in the O_2_ and water vapour traces. Oxygen consumption was calculated using eqn 10.1 of Lighton (2019):(1)
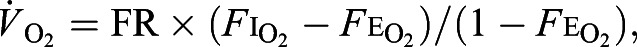
where FR is the incurrent flow rate (ml min^−1^), *F*i_O_2__ is the incurrent fractional O_2_ concentration (0.2095) and *F*e_O_2__ is the excurrent fractional O_2_ concentration. At each *T*_a_, we measured resting values of *V̇*_O_2__, WVP and *T*_b_ using the mean of the most stable 5 min period of *V̇*_O_2__. For each *T*_a_, we excluded data from any bird that did not remain calm based on our observations of their behaviour, which we later verified using our video recordings of the experiments. Birds were assumed to be post-absorptive as murres digest food within 1–2 h (Gaston and Noble, 1985) and had fasted in the chamber for 14.65±1.4 h prior to starting our heat tolerance runs. Therefore, to transform *V̇*_O_2__ to RMR (W), we used eqn 9.13 of Lighton (2019) to derive energy equivalents (J ml^−1^ O_2_), assuming a respiratory quotient of 0.71 (Walsberg and Wolf, 1995). We calculated rates of EWL (mg min^−1^) by converting WVP (kPa) into water vapor density (mg ml^−1^) using the following equation from the Sable Systems water vapor analyser manual, then multiplying by the incurrent flow rate:(2)
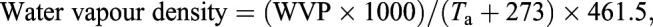
where 461.5 (J kg K^−1^) is the individual gas constant for water vapor. To determine how efficient an individual murre was at dissipating heat, we calculated their evaporative cooling efficiency by converting rates of EWL into EHL (W) assuming 2.406 J mg^−1^ H_2_O, and dividing by MHP, with low EHL/MHP values assumed to indicate a lower ability to dissipate heat (Lasiewski et al., 1966).

We characterized the onset of heat stress as the *T*_a_ inflection point for *T*_b_, RMR, EWL and EHL/MHP in murres, obtained by fitting broken-stick regressions to identify significant changes in slope, using the R package *SiZer* (https://cran.r-project.org/package=SiZer). To examine the effect of body mass (*M*_b_, measured before heat tolerance runs) and *T*_a_ on *T*_b_, RMR, EWL and EHL:MHP, we took a subset of the data at the inflection points and fitted linear mixed effect models on the data below and above the inflection points using the lme4 package in R (Bates et al., 2015). We built a global model with *M*_b_, *T*_a_ and their two-way interaction as predictors. To account for repeated measurements on the same individual, we included individual bird identification as a random factor. We then performed model selection using the dredge function in the *MuMIn* package (https://CRAN.R-project.org/package=MuMIn) based on Akaike's information criterion adjusted for small sample size (AICc). The minimum adequate model within a ΔAICc<2 was considered the best model (Burnham and Anderson, 2002). We calculated AICc weights based on all available models. We used paired *t*-tests to compare the *M*_b_ of birds before and after our heat tolerance runs.

Each of the top models met assumptions for normality, linearity and homogeneity of variance. We tested for outliers by calculating Cook's distance values for each bird using the *influence.ME* package (Nieuwenhuis et al., 2012). As the model for EWL above the inflection point for *T*_a_ had one individual with a Cook's distance value >1 (Logan, 2010), we fitted a robust-mixed effect model to the data for this particular model using the *robustlmm* package (Koller, 2016). All analyses were run using R 3.6.3 (http://www.R-project.org/) and significance was judged at α=0.05. We made all figures using *ggplot2* (Wickham, 2016)*.* Data are reported as means±1 s.e.m. All raw data are tabulated in Table S1.

## RESULTS

### *M*_b_

**Fig. 1.**
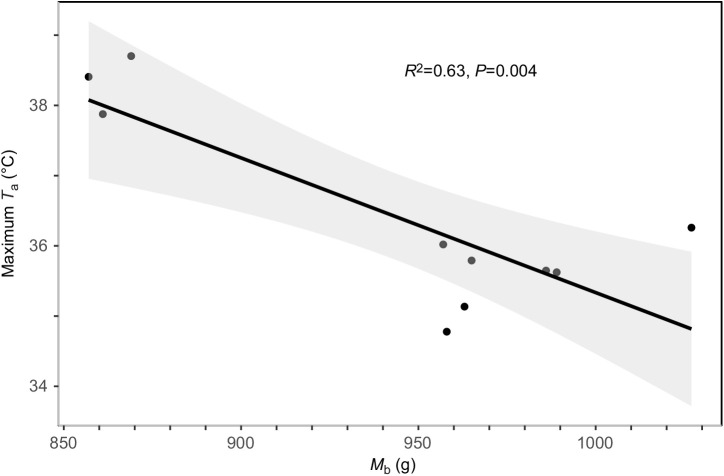
**Linear regression of the relationship between maximum tolerated air temperature (*T*_a_) and total body mass (*M*_b_) prior to heat tolerance runs.** Data are for 10 murres. The shaded area represents the 95% confidence interval (CI) around the predicted values.

Murres weighed 999.3±20.2 g at capture (*M*_b_ range 906–1079 g, *n*=10). *M*_b_ decreased (paired *t*-test, *t*_9_=−12.30, *P*<0.0001) after overnight fasting in the thermoneutral zone (mean 14.65 h) prior to heat tolerance runs. Mean *M*_b_ prior to heat tolerance trials was 943.2±18.8 g and declined significantly during runs (post-heat tolerance *M*_b_=900.7±18.0 g; paired *t*-test, *t*_9_=−21.91, *P*<0.0001), with a mean loss of 42.5±1.9 g (i.e. approximately 4.5% loss). When we compared the maximum *T*_a_ tolerated by murres with *M*_b_ prior to heat tolerance trials, larger birds had lower heat tolerance limits, with maximum tolerated *T*_a_ decreasing with increasing *M*_b_ (Fig. 1; *r*^2^=0.63, *F*_1,8_=16.5, *P*=0.0036).

### *T*_b_

**Fig. 2.**
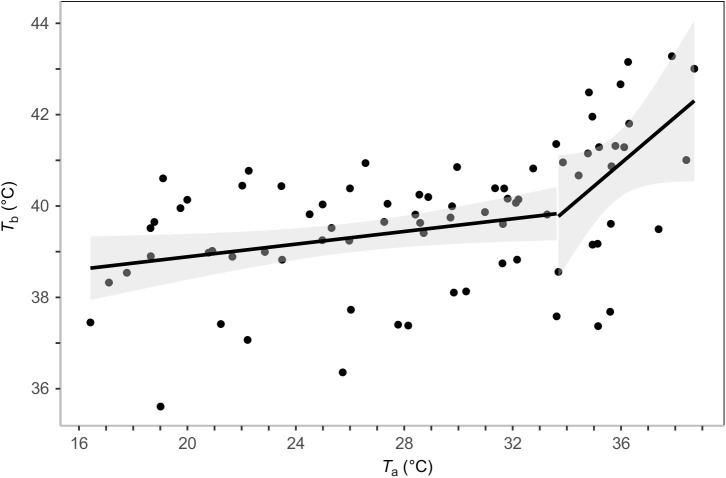
**Linear regression of the relationship between body temperature (*T*_b_) and *T*_a_.** *T*_b_ data are for 10 murres (*n=*78). A significant inflection point in *T*_b_ was identified at *T*_a_=33.7°C. The shaded area represents the 95% CI around the predicted values.

During heat tolerance trials, *T*_b_ of murres ranged from 35.6°C at *T*_a_=19.0°C to 43.3°C at *T*_a_=37.9°C. Murres demonstrated a significant inflection point in *T*_b_ at *T*_a_=33.7°C (Fig. 2; 95% confidence interval CI=22.2–37.0°C). Below the inflection point, the minimum adequate model explaining *T*_b_ included *T*_a_, *M*_b_ and their interaction (Table 1). Above the inflection point, the minimum adequate model included *T*_a_ only, with *T*_b_ increasing with *T*_a_ (Table 2; Fig. 2). *T*_b_ increased from 38.6±0.5°C at *T*_a_=18.8±0.4°C to a mean maximum of 41.4±0.5°C at *T*_a_=36.5±0.5°C.

**Table 1.**
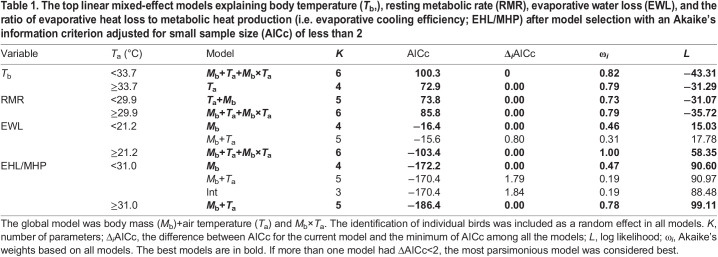
The top linear mixed-effect models explaining body temperature (*T*_b_,), resting metabolic rate (RMR), evaporative water loss (EWL), and the ratio of evaporative heat loss to metabolic heat production (i.e. evaporative cooling efficiency; EHL/MHP) after model selection with an Akaike's information criterion adjusted for small sample size (AICc) of less than 2

**Table 2.**
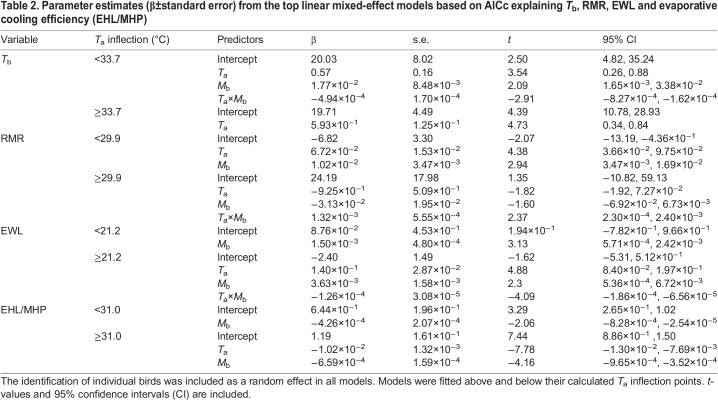
Parameter estimates (β±standard error) from the top linear mixed-effect models based on AICc explaining *T*_b_, RMR, EWL and evaporative cooling efficiency (EHL/MHP)

### RMR

**Fig. 3.**
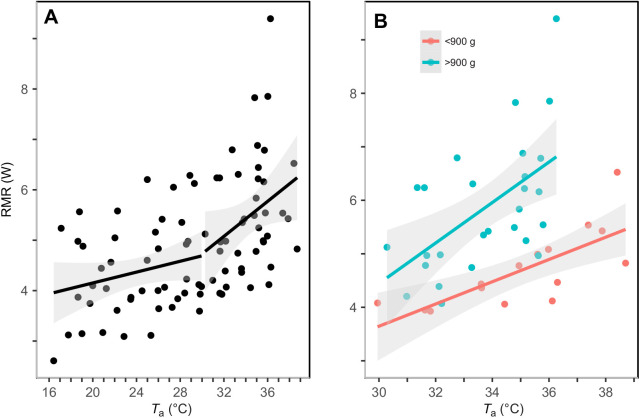
**Linear regression of the relationship between resting metabolic rate (RMR) and *T*_a_.** RMR data are for 10 murres. (A) A significant inflection point in RMR was identified at *T*_a_=29.9°C (*n=*85). (B) Data for birds with *M*_b_ greater (*n*=27) or lower (*n*=15) than 900 g at *T*_a_≥29.9°C. The shaded area represents the 95% CI around the predicted values.

RMR ranged from 2.6 W at *T*_a_=16.4°C to 9.4 W at *T*_a_=36.3°C. A significant inflection point in RMR occurred at *T*_a_=29.9°C (Fig. 3A; 95% CI=18.8–34.1°C). Below the inflection point, the minimum adequate model explaining RMR included *T*_a_ and *M*_b_ (Table 1), and RMR increased with both *T*_a_ (Table 2; 0.07±0.02 W °C^−1^) and *M*_b_ (0.01±0.003 W g^−1^). Above the inflection point, the minimum adequate model included *T*_a_, *M*_b_ and the interaction between *T*_a_ and *M*_b_. To control for the interaction, we divided individual birds into two *M*_b_ categories based on Fig. 1 (above and below 900 g), and found RMR increased with *T*_a_ at a faster rate (Fig. 3B; 0.35±0.06 W °C^−1^, 95% CI=0.24–0.47) in murres that were larger than 900 g, relative to smaller birds (0.22±0.04 W °C^−1^, 95% CI=0.15–0.29). Across all birds, RMR increased from 3.97±0.3 W at *T*_a_=18.8±0.4°C to a mean maximum of 6.24±0.4 W at *T*_a_=36.4±0.4°C.

### EWL

**Fig. 4.**
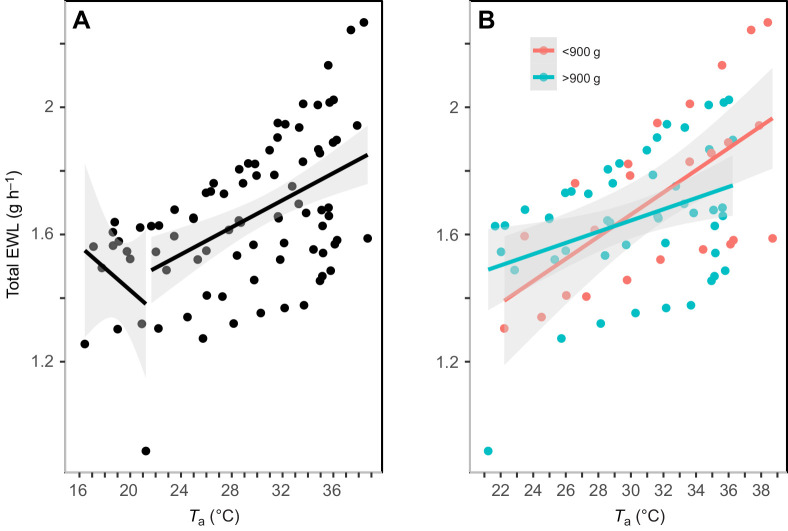
**Linear regression of the relationship between evaporative water loss (EWL) rate and *T*_a_.** EWL data are for 10 murres. (A) A significant inflection point in EWL was identified at *T*_a_=21.2°C (*n=*85). (B) Data for birds with *M*_b_ greater (*n*=49) or lower (*n*=24) than 900 g at *T*_a_≥21.2°C. The shaded area represents the 95% CI around the predicted values.

EWL rates ranged from 0.92 g h^−1^ at *T*_a_=21.2°C to 2.27 g h^−1^ at *T*_a_=38.4°C. Murres began panting at *T*_a_=25.9±0.5°C and *T*_b_=39.3±0.5°C. We detected a significant inflection point in rates of EWL at 21.2°C (Fig. 4A; 95% CI=20.9–36.6°C). However, one bird displayed noticeably lower rates of EWL relative to the others and the removal of this bird increased the EWL inflection point to *T*_a_=24.5°C (95% CI=17.7–36.8), a value closely aligning with the mean *T*_a_ when panting started. Below the first inflection point, EWL rates were best predicted by *M*_b_ (Table 1). Above the inflection point, the minimum adequate model explaining rates of EWL included *T*_a_, *M*_b_ and the interaction between *T*_a_ and *M*_b_ (Table 2; Fig. 4B). To control for the interaction, we divided individual birds into two *M*_b_ categories based on Fig. 1 (above and below 900 g). EWL increased with *T*_a_ at a faster rate (Fig. 4B; 0.04±0.005 g h °C^−1^, 95% CI=0.03–0.05) in murres that were smaller than 900 g relative to larger birds (0.02±0.002 g h °C^−1^, 95% CI=0.01–0.02). Murres increased their rate of EWL 1.26-fold relative to baseline rates measured at *T*_a_=18.8±0.4°C, increasing from a mean of 1.44±0.1 g h^−1^ to 1.80±0.1 g h^−1^ at *T*_a_=36.4±0.4°C.

### Evaporative cooling efficiency (EHL/MHP)

**Fig. 5.**
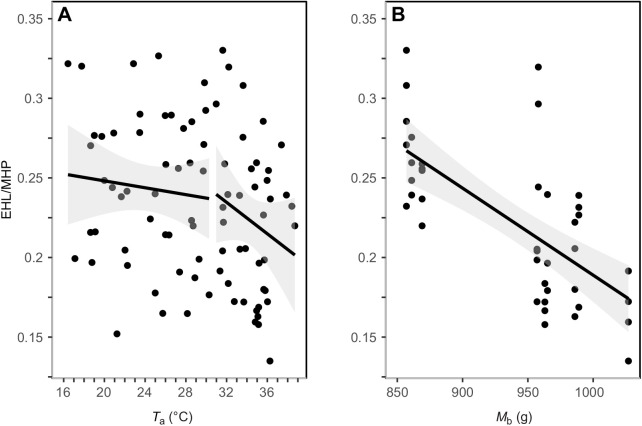
**Linear regression of the relationship between evaporative cooling efficiency and *T*_a_.** Evaporative cooling efficiency data (the ratio of evaporative heat loss to metabolic heat production, EHL/MHP) are for 10 murres. (A) A significant inflection point in EHL/MHP was identified at *T*_a_=31.0°C (*n=*85). (B) EHL/MHP versus *M*_b_ at *T*_a_≥31.0°C. The shaded area represents the 95% CI around the predicted values.

Evaporative cooling efficiency (EHL/MHP) ranged from 0.33 at *T*_a_=31.6°C to 0.13 at *T*_a_=36.3°C (mean±s.e.m.=0.23±0.01). A significant inflection point was detected at *T*_a_=31.0°C (Fig. 5A; 95% CI=18.6–35.2°C). Below the inflection point, EHL/MHP was best predicted by *M*_b_ (Table 1). Above the inflection point, the minimum adequate model explaining EHL/MHP included *T*_a_ and *M*_b_ (Fig. 5B). EHL/MHP decreased significantly with both *T*_a_ (Table 2; −0.01±0.001 g h °C^−1^, 95% CI=−0.013 to −0.008) and *M*_b_ (Table 2; −0.0007±0.0002 g h g^−1^, 95% CI=−0.001 to −0.0003).

## DISCUSSION

Murres displayed markedly reduced heat tolerance relative to birds originating from hot and arid climates, with individuals showing signs of active physiological responses to evaporate heat at much milder temperatures. Murres, to our knowledge, also have the lowest maximum evaporative cooling efficiency ever recorded in birds, including Cape rockjumpers (*Chaetops frenatus*, ∼0.75; Oswald et al., 2018) and the juniper titmouse (*Baeolophus ridgwayi*, ∼0.75; Weathers and Greene, 1998). Below, we outline how murre physiological responses to increasing *T*_a_ demonstrate their limited capacity to tolerate even moderately elevated temperatures, making them vulnerable to Arctic warming when incubating on sun-exposed cliffs.

### *T*_b_

When *T*_b_ exceeds *T*_a_, facultative hyperthermia (Gerson et al., 2019; Tieleman and Williams, 1999) aids birds in conserving water by increasing the thermal gradient between *T*_b_ and *T*_a_ (Weathers, 1981; Tieleman and Williams, 1999; Gerson et al., 2019). Murres displayed hyperthermia and increased their *T*_b_ at *T*_a_=33.7°C (95% CI=22.2–37.0°C). The inflection point at *T*_a_=33.7°C is similar to that in snow buntings (*Plectrophenax nivalis*, 32.6°C, 95% CI=31.0–34.4°C; O'Connor et al., 2021), the only other Arctic bird for which heat tolerance measurements are available, and is also within the *T*_a_ range of desert and non-desert avian species (Tieleman and Williams, 1999). Murres' maximum *T*_b_ (43.3°C) was similar to the mean *T*_b_ of birds from non-desert (43.3°C, range 41.1–45.8°C) and desert (43.6°C, range 41.5–45.4°C) environments at *T*_a_=45.0°C (Tieleman and Williams, 1999). While the mean increase in *T*_b_ across 23 desert and non-desert avian species from their lower critical temperatures to 45.0°C was similar to that of murres (3.3 versus 2.8°C), the maximum *T*_a_ endured by any murre was much lower (38.7 versus 45.0°C; Tieleman and Williams, 1999). The regulation of a low *T*_b_ will result in a narrowing thermal gradient as *T*_a_ increases, impeding murres' capacity for dry heat loss and forcing them to increasingly rely on evaporative cooling.

### RMR

Murres increased their RMR at an upper critical temperature (*T*_uc_) of 29.9°C (95% CI=18.8–34.1°C), which is similar to the *T*_uc_ of other cold-region birds, including snow buntings (29.8°C, 95% CI=27.9–42.2°C; O'Connor et al., 2021), little penguins (*Eudyptula minor*, 30.0°C; Stahel and Nicol, 1982), Peruvian penguins (*Spheniscus humboldti*, 30.0°C; Drent and Stonehouse, 1971) and Cassin's auklet (*Ptychoramphus aleuticus*, ∼25.0°C; Richman and Lovvorn, 2011) and lower than those of arid and semi-arid birds (33.9–46.5°C; McKechnie et al., 2017; Smith et al., 2015; Smith et al., 2017). RMR increased by 1.57-fold from a mean *T*_a_=18.8°C to 36.4°C in murres, which is higher than the mean fractional increase in snow buntings (1.4; O'Connor et al., 2021) and some desert birds (1.26–2.66; McKechnie et al., 2016, 2017, Smith et al., 2017, McWhorter et al., 2018, Talbot et al., 2018, Czenze et al., 2020). When we compared mass-specific RMRs in murres above their *T*_uc_, the slope was approximately twice as steep as that predicted from their mean *M*_b_ (0.298 versus 0.146 mW g °C^−1^; Weathers, 1981). In addition, post-absorptive RMRs of murres (Table 3) are approximately 1.1–2.3 times higher than predicted by allometric equations for non-passerines and seabirds (Aschoff and Pohl, 1970; Croll and McLaren, 1993; Ellis, 1984; Gabrielsen and Ellis, 2002; Lasiewski and Dawson, 1967). Murres' high RMRs at thermoneutral temperatures are proposed to be an adaptive response to higher energy requirements in cold climates, diving foraging strategies and high activity levels (Croll and McLaren, 1993; Ellis, 1984; Gabrielsen and Ellis, 2002; Gabrielsen et al., 1988). However, high RMRs will likely become disadvantageous at higher air temperatures because they will lead to murres experiencing greater total heat loads, which must ultimately be dissipated.

**Table 3.**
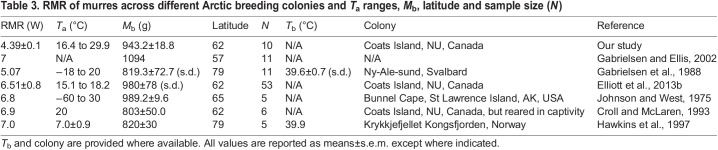
RMR of murres across different Arctic breeding colonies and *T*_a_ ranges, *M*_b_, latitude and sample size (*N*)

### EWL

Murres began to show signs of heat stress, such as panting and increased rates of EWL, at relatively low air temperatures. In comparison, the onset of panting and increased EWL rate occurred at higher air temperatures in snow buntings (33.2°C and 34.6°C, 95% CI=31.1–36.2°C, respectively; O'Connor et al., 2021) and arid-zone birds (panting: 38.0–42.3°C, increase in EWL: 36–46.5°C; Smith et al., 2015, 2017, McKechnie et al., 2017, O'Connor et al., 2017, Czenze et al., 2020). It is worth noting that a *T*_a_ of 21.2°C is ecologically relevant, and corresponds to the maximum *T*_a_ at Coats Island during years in which murres displayed noticeable heat dissipation behaviours and experienced higher rates of mortality and egg loss due to the combination of heat stress and mosquito parasitism (22°C in 1998: Gaston et al., 2002; 21.2 and 21.3°C in 2011: Gaston and Elliott, 2013).

The maximum *T*_a_ tolerated was correlated with higher evaporative scope (maximum EWL/minimum EWL) in birds (Czenze et al., 2020). The evaporative scope of murres was low relative to that of snow buntings (O'Connor et al., 2021) and birds from desert environments (range 4.73–15.33; McKechnie et al., 2017, Smith et al., 2017, McWhorter et al., 2018, Czenze et al., 2020). The murres' low evaporative scope presumably corresponded with their low mean maximum *T*_a_ (36.4°C) relative to that of other birds (snow buntings: 43.0°C: O'Connor et al., 2021; desert birds: 46.0–54°C: McKechnie et al., 2017; Smith et al., 2017; McWhorter et al., 2018; Czenze et al., 2020). At 25°C, the mean EWL in murres was 1.54 g h^−1^ or approximately 37.0 ml day^−1^, higher than the allometric prediction (31.2 ml day^−1^; see eqn 2 of Williams, 1996). Similarly, the EWL rate of murres at 25°C was higher than that (25.8 ml day^−1^) of Houbara bustards (*Chlamydotis macqueenii*), a similar-sized (1245 g) desert bird (Tieleman et al., 2002). Furthermore, based on equations from McKechnie and Wolf (2010), the predicted EWL inflection point was higher (33.6°C; but within the 95% CI=20.9–36.6°C), and the predicted mass-specific slope in the rates of EWL with *T*_a_ was 16.6-fold steeper than observed in murres (0.479 versus 0.029 mg H_2_O g h °C^−1^). The minimal increase in EWL rate of murres suggests they are restricted in their capacity to increase evaporative heat dissipation with increasing temperatures.

### Evaporative cooling efficiency (EHL/MHP)

To our knowledge, murres displayed the lowest maximum evaporative cooling efficiency ever reported in a bird. However, we acknowledge that our dew points, and resulting absolute humidity levels, exceed those of previous heat tolerance investigations (e.g. dew points <5°C; Smith et al., 2017). Consequently, the maximum evaporative cooling capacity of thick-billed murres may have been negatively impacted given the interactive effects of humidity and *T*_a_ on EHL/MHP (Lasiewski et al., 1966). Unfortunately, available data on the interaction between humidity and *T*_a_ in birds at high *T*_a_ is scarce and suggests substantial variation among taxa (e.g. Gerson et al., 2014; van Dyk et al., 2019), and we therefore at present cannot definitively say whether our dew points markedly influenced murres' cooling efficiency. At best, murres could only dissipate one-third of the heat they produced metabolically. Ratios of EHL/MHP<1 indicate an organism is unable to dissipate all of its metabolic heat through evaporative heat loss (Lasiewski et al., 1966). While snow buntings also showed low evaporative cooling efficiencies, the inflection point for *T*_a_ in murres was lower than in buntings (31°C, 95% CI=18.6–35.2°C versus 36.7°C, 95% CI=31.0–42.3°C; O'Connor et al., 2021). More importantly, evaporative cooling efficiency decreased with increasing *T*_a_ in murres, suggesting they were producing heat at a faster rate than they were dissipating it. Murres depend on metabolic heat production to maintain their core *T*_b_ (Johnson and West, 1975) and increase their metabolic rate with decreasing water temperatures (Croll and McLaren, 1993). High RMRs and low increases in EWL likely resulted in very low EHL/MHP values and heat tolerance capacities in murres.

### *M*_b_ and heat tolerance

Larger murres were more vulnerable to heat stress as a result of higher RMRs and lower rates of EWL. Foraging strategies of murres vary with body size, with larger murres spending the most time at deeper and colder depths (Orben et al., 2015). While a larger body size may convey an advantage for minimizing heat loss in murres when diving in cold water, it may also result in an increased risk of overheating while sitting on their nesting ledges, as evaporative cooling efficiency and heat tolerance limits both declined with increasing *M*_b_. To our knowledge, this is the first heat tolerance study on a diving seabird, or any large polar bird, and the adaptations for diving in icy waters may conflict with murres' ability to tolerate heat. In contrast, heat tolerance limits increased with *M*_b_ in Australian passerines (McKechnie et al., 2017), but there was no clear relationship in Sonoran passerines (Smith et al., 2017). Body mass was the most important predictor of EWL across 174 bird species, with higher EWL rates in larger birds (Song and Beissinger, 2020); however, smaller passerines experience higher rates of mass-specific EWL rates and have a greater risk of dehydration than larger birds (Albright et al., 2017; McKechnie and Wolf, 2010). Larger murres demonstrated steeper increases in RMR and shallower increases in EWL, which clearly influenced EHL/MHP. In contrast, maximum EHL/MHP increased with increasing *M*_b_ in Australian passerines (McKechnie et al., 2017); however, other studies have not found a clear relationship between *M*_b_ and EHL/MHP (Smith et al., 2017; Whitfield et al., 2015).

### Conclusions and ecological implications

Recent heat waves in the Gulf of Alaska were associated with the mass mortality and reproductive failures of several colonies of common murres (*Uria aalge*) (Piatt et al., 2020). While these mortalities were hypothesized to be due to an ‘ectothermic vice’ on forage fish where birds faced increased foraging competition and reduced prey quality and quantity (Piatt et al., 2020), direct effects of heat stress may have also contributed. We demonstrated that thick-billed murres have limited heat tolerance. As a dark-plumage bird with 12–24 h incubation shifts, low heat tolerance may explain their heat dissipating behaviours, reproductive failures and mortalities at a maximum *T*_a_ as low as 16–22°C (Gaston and Elliott, 2013; Gaston et al., 2002). Importantly, when incubating in full sun, murre surface temperatures can reach 46°C (Gaston et al., 2002); therefore, the operative temperatures of the birds are likely much higher than the maximum *T*_a_ measured at Coats Island. In addition, the maximum EWL rate (2.27 g h^−1^) recorded here would only result in a loss of 2.9% of the mean *M*_b_ (943 g) of murres over the period of an incubation shift (12 h, most of which may not be in direct sunlight because of cloud cover and nest orientation on the cliff), and is likely unable to cause dehydration. Thus, we argue that murre mortality is likely due to tissue stress associated with high body temperatures coupled with blood loss from mosquito parasitism (Gaston and Elliott, 2013; Gaston et al., 2002).

While we used multiple measures, we acknowledge that our low sample size of individual murres (*n=*10) due to logistical constraints makes it difficult for us to draw solid conclusions. Our confidence intervals surrounding our inflection point temperatures were large (approximately 15°C), which may be the result of our small sample size and large range of *M*_b_ (857–1027 g) among individuals. While we measured the effects of warmer temperatures on resting murres, further investigation should consider the impacts of heat stress during high energy behaviours, as endotherms are limited in the maximal energy they can expend by their ability to dissipate heat (Speakman and Król, 2010). For example, common eiders (*Somateria mollissima*) experience hyperthermia during flight and must stop to cool down, which is a major cost of migration (Guillemette et al., 2016, 2017). As murres are large seabirds with high energetic costs of flight (Elliott et al., 2013b) and high daily energy requirements (Elliott and Gaston, 2014), their low heat tolerance may lead to energetic trade-offs to support their high costs of thermoregulation, which may impact their behaviour, reproductive success and, ultimately, survival.

## Supplementary Material

Supplementary information
