# Supplementary material for: Limited heat tolerance in a cold-adapted seabird: implications of a warming Arctic
Source: J Exp Biol. 2021 Jul 7;224(13):jeb242168. doi: 10.1242/jeb.242168 (PMC8278010; doi:10.1242/jeb.242168)
Supplement: Supplementary information [file jexbio-224-242168-s1.pdf]

Table S1. Raw data values of physiological traits measured for heat tolerance [body temperature (T_b), resting metabolic rate (RMR), evaporative water loss (EWL), ratio of evaporative heat loss to metabolic heat production (EHL/MHP)] and their associated air temperatures (T_a) for thick-billed murres (*n*= 10). The total body mass of murres was measured before (mass 1) and after (mass 2) each heat tolerance run.

Click here to download Table S1
